# InterPepScore: a deep learning score for improving the FlexPepDock refinement protocol

**DOI:** 10.1093/bioinformatics/btac325

**Published:** 2022-05-16

**Authors:** Isak Johansson-Åkhe, Björn Wallner

**Affiliations:** Division of Bioinformatics, Department of Physics, Chemistry and Biology, Linköping University, SE-581 83 Linköping, Sweden; Division of Bioinformatics, Department of Physics, Chemistry and Biology, Linköping University, SE-581 83 Linköping, Sweden

## Abstract

**Motivation:**

Interactions between peptide fragments and protein receptors are vital to cell function yet difficult to experimentally determine in structural details of. As such, many computational methods have been developed to aid in peptide–protein docking or structure prediction. One such method is Rosetta FlexPepDock which consistently refines coarse peptide–protein models into sub-Ångström precision using Monte-Carlo simulations and statistical potentials. Deep learning has recently seen increased use in protein structure prediction, with graph neural networks used for protein model quality assessment.

**Results:**

Here, we introduce a graph neural network, InterPepScore, as an additional scoring term to complement and improve the Rosetta FlexPepDock refinement protocol. InterPepScore is trained on simulation trajectories from FlexPepDock refinement starting from thousands of peptide–protein complexes generated by a wide variety of docking schemes. The addition of InterPepScore into the refinement protocol consistently improves the quality of models created, and on an independent benchmark on 109 peptide–protein complexes its inclusion results in an increase in the number of complexes for which the top-scoring model had a DockQ-score of 0.49 (Medium quality) or better from 14.8% to 26.1%.

**Availability and implementation:**

InterPepScore is available online at http://wallnerlab.org/InterPepScore.

**Supplementary information:**

Supplementary data are available at *Bioinformatics* online.

## 1 Introduction

Interactions between a protein receptor and a smaller flexible peptide fragment make up 15–40% of all protein–protein interactions (Petsalaki and Russell, 2008), and are involved in vital cell functions such as cell life-cycle regulation (Midic *et al.*, 2009). The flexibility of the peptide and often transient nature of the interaction (Tu *et al.*, 2015) makes them difficult to study experimentally. Thus, computational protein–peptide docking methods are needed to understand the molecular mechanisms and details of the interactions (Helander *et al.*, 2015; Petsalaki and Russell, 2008; Wei *et al.*, 2019).

The approaches to solving the peptide–protein docking problem ranges from advanced searches for structural templates of interaction such as with InterPep2 (Johansson-Åkhe *et al.*, 2020) or SPOT-peptide (Litfin *et al.*, 2019), to exhaustive sampling of docked conformations as in PIPER-FlexPepDock(Alam *et al.*, 2017) or pepATTRACT (Schindler *et al.*, 2015), and end-to-end machine-learning generated models of interaction as with the input adapted AlphaFold protocols (Tsaban *et al.*, 2022) or AlphaFold-Multimer (Evans *et al.*, 2021). Common to most of the docking approaches is the need for a final step of all-atom refinement to optimize the final structures. Several of the methods above use the Rosetta FlexPepDock refinement protocol (Raveh *et al.*, 2010) for this final step.

Neural networks have dominated the fields of protein structure quality assessment and tertiary structure prediction lately (Kryshtafovych *et al.*, 2019; Pereira *et al.*, 2021), with neural network architectures moving away from the generalized solutions based on ideas from image recognition and toward specialized architectures for the protein structure problem, like AlphaFold2 which combines attention layers, triangle multiplicative updates for graph inference and 3D-equivariant transformers to go from sequence alignment to structure prediction (Jumper *et al.*, 2021). To describe molecules and protein structures as graphs is an intuitive representation. Graph neural networks and graph convolution networks have both seen use and great success in areas such as function prediction (Gligorijevic *et al.*, 2019), protein–protein interaction site prediction (Fout *et al.*, 2017) and tertiary structure quality assessment (Baldassarre *et al.*, 2021).

Previously, we developed InterPepRank, a small graph convolution network, for coarse-grained peptide–protein complex structure quality assessment using a limited vertex and edge-space, trained on rigid-body-docked structures (Johansson-Åkhe *et al.*, 2021). Here, we introduce the InterPepScore scoring term for use with the Rosetta FlexPepDock refinement protocol by continually predicting DockQ score (Basu and Wallner, 2016) during FlexPepDock Monte Carlo sampling. InterPepScore utilizes a network architecture similar to that of InterPepRank, but includes learnable edge- and global features with a more complex information-passing scheme through the graph, as well as an unlimited vertex- and edge-space, to better tackle the more detail-oriented refinement problem. When InterPepScore is added as an additional scoring term, it consistently improves the performance by increasing the quality of the refined models, and will as such improve any docking pipeline using FlexPepDock for its final refinement.

## 2 Methods and development

### 2.1 Dataset

To train and benchmark the method, 6857 peptide–protein complexes from the PDB (Berman *et al.*, 2000) (December 12, 2018) were redundancy reduced down to 684 complexes by clustering at 30% sequence identity. In this case, a peptide–protein complex was considered any protein–protein complex where one ‘receptor’ chain was at least 50 residues in length and shared at least 200 Å^2^ of contact surface with a ‘peptide’ chain of less than or equal to 25 residues. Complexes with post-translational modifications of the peptide or interacting interface were not included in the dataset. All other post-translational modifications of the receptor structures were reset to the unmodified residues.

A set of 109 complexes were selected as a test set and a separate set of 95 complexes selected as validation set. The sets were generated randomly, but if any receptor of any set shared a CATH superfamily annotation with any other set, the randomization was repeated until the sets shared no such connections. In the case that a receptor had no CATH annotation, it was awarded the same annotation as the chain in the full non-redundancy-reduced set that it matched to with the largest TM-score when aligned with TM-align (Zhang and Skolnick, 2005).

The training set was constructed by including every complex from the initial non-redundancy reduced set which did not share a CATH superfamily annotation with neither the test set nor the validation set, resulting in 4447 peptide–protein complexes for training, evenly distributed between the three primary CATH classes.

### 2.2 Generating initial starting structures

The purpose of InterPepScore is to aid in Rosetta scoring of peptide–protein complexes during refinement by guiding the refinement process toward more native-like structures. Thus, the training data should consist of several snapshots from the FlexPepDock refinement processes, *trajectories* of peptide refinement starting from models of varying quality.

The initial models used as starting points for the FlexPepDock trajectories for training and validation as well as the models to refine for testing and benchmarking were generated using three different schemes: (i) perturbation of the native peptide similarly to the original FlexPepDock refinement paper (Raveh *et al.*, 2010), (ii) rigid-body-docking with several sampled peptide conformations similarly to PIPER-FlexPepDock (Alam *et al.*, 2017) and (iii) template-based docking similarly to InterPep2 (Johansson-Åkhe *et al.*, 2020).

For the test and validation sets, each scheme for generating starting points contributed with four starting models, in total 12 starting points per complex for all three schemes.

For the training set, a total of 440 000 starting points were generated evenly distributed between CATH superfamilies in the set, to not bias the training set toward particular folds. Half of all starting points were generated by rigid-body-docking, and the remaining were evenly distributed between perturbation from the native fold and template-based docking, respectively.

The sets would need to contain both possible to refine (already close but not identical to the native structure) and difficult to refine (further from native structure) starting positions, optimally evenly distributed between different CATH superfamilies and starting point generation schemes so as not to bias the trained InterPepScore. To ensure sufficient number of starting points close to the correct structure to enable refinement, for each complex, exactly half of all starting points from each generation scheme were forced to have an LRMSD < 5.5 Å or any contiguous stretch of at least half the peptide (or 5 residues, whichever is larger) with an LRMSD < 4.0 Å. The first cutoff was chosen as it is the limit for salvageable decoys from FlexPepDock protocol paper (Raveh *et al.*, 2010). For longer peptides, sometimes more of the peptide is modeled than what is actually in contact with the receptor, which is why the second cutoff was also used. The forced selection was obtained by continually discarding generated positions and generating new ones until the thresholds were satisfied. Starting positions satisfying these criteria are referred to as ‘salvageable’. This resulted in half of all starting positions for each complex having high LRMSD and half having low, and the distribution of high versus low starting positions were the same for each complex for each generation scheme. As each CATH superfamily contributed an equal amount of poses to the set, there should be no bias in the training set toward any specific CATH superfamily or refinement difficulty.

Details for how starting points were generated using the different schemes can be found below:

- Perturbation of the native position: PyRosetta was used to apply random shear, small, rigid-transformation and rigid-rotation movements to the native peptide. In this starting point generation scheme, the magnitude of the changes applied were modified after each starting point generated in accordance with if that point became salvageable or unsalvageable (increased movement sizes if the point became salvageable and vice versa).
- Rigid-body docking: Rigid configurations of the peptide was generated as in the PIPER-FlexPepDock paper and docked with PIPER on the receptor surface (Alam *et al.*, 2017). The best-scoring decoys by PIPER score were selected.
- Template-based docking: The receptor was aligned by TM-align to every protein–protein complex in the PDB (Zhang and Skolnick, 2005). The same rigid configurations for the peptide as used for the rigid-body docking were structurally aligned to the other side of the interface for the best scorers by use of InterComp (Mirabello and Wallner, 2018).

In separate tests, structures generated by AlphaFold-Multimer-v1 (Evans *et al.*, 2021) are refined. These structures are generated by running AlphaFold-Multimer with standard settings while disallowing templates.

### 2.3 Graph network architecture

InterPepScore is a global score implemented as a graph network with protein residues as vertices and edges between residues within 10 Å (as defined between their C_β_, or C_α_ in case of Glycine) of each other. The 10 Å cutoff was selected as such a graph is already calculated as part of the Rosetta scoring process when any context-dependent two-body energy is included in the scoring function (Leaver-Fay *et al.*, 2011) and that contact distance threshold has previously been proven adequate for global protein prediction metrics (Baldassarre *et al.*, 2021). The features of the edges denote if the residues are covalently bound or not and if the edges are self-edges, similarly to previous work (Johansson-Åkhe *et al.*, 2021). The vertex features are BLOSUM62 columns representing the amino acid residues and one-hot identifiers if the residues belong to the protein or peptide chain. BLOSUM62 matrix columns were used instead of learning a sequence embedding given the rather small number of unique sequences in the training set. Pre-trained sequence embeddings, such as Bepler (Bepler and Berger, 2019) and ProtBert (Elnaggar *et al.*, 2020) were tried but did not improve performance (Supplementary Table S1), most likely because the training set is too small to cope with the large dimensions of the embeddings (121 and 1024, respectively). Multiple sequence alignments were not used to ensure InterPepScore can be used as any other Rosetta scoring term without additional pre-calculation of features.

**Fig. 1.**
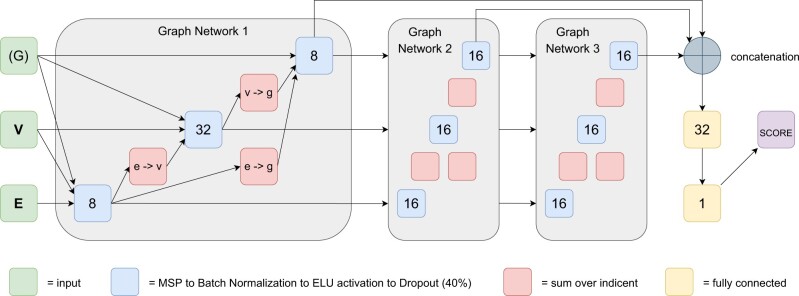
The neural network architecture of InterPepScore. E, V and G represents edge features, vertex features and global features, respectively. The blue boxes with numbers in the graph network blocks represent the core learning elements of the network. The numbers inside boxes indicate the length of the feature vector they output. Note that in a graph network, the V row always outputs one feature vector *per vertex*. Similarly, there is one feature vector per edge. The global input G is in parenthesis as InterPepScore only uses a dummy variable as the global input, but after the first graph network layer a global representation is learned which is then used for later layers, as seen in the figure (A color version of this figure appears in the online version of this article.)

Three layers of graph network forward passes—updating all vertex, edge and global features with ELU activation as implemented through the Graph Nets package (Battaglia *et al.*, 2018) followed by a final convolution of the learned global representations make up the architecture of the network, see Figure 1. Note that InterPepScore does not use any global input features, but only vertex and edge features with a constant dummy-variable placeholder for global input features. A global representation is learned after each graph net layer. During training, dropout and batch normalization were used. Each learning component block of the *Graph Network* (blue blocks in the figure) are composed of the following, in feed-forward order:

- Concatenation: Concatenation of the feature vectors of all incident elements and the active elements’ own feature vectors. In cases where the number of incident elements is unknown, the blue boxes are always preceded by a red box, which indicates a summation of the feature vectors of the incident elements. The global feature vector is incident to all other elements.
- Perceptron: The basic learning component is a simple linear perceptron.
- Batch normalization
- ELU activation
- Dropout: During training, a 40% dropout rate of features was employed.

### 2.4 InterPepScore training

InterPepScore was implemented using pyRosetta (Chaudhury *et al.*, 2010) and was trained and validated on Rosetta FlexPepDock simulation trajectories, including both accepted and rejected structures during the Monte Carlo sampling steps. S-score normalized LRMSD was used as the target function with a *d*_0_ of 8.0, LRMSD_norm_ = 1/(1 + (LRMSD/8.0)^2^). A global loss for the peptide as a whole was used rather than a per-residue loss as even if a few peptide residues are positioned roughly correctly, this does not mean they are rotated or angled so as to allow the rest of the peptide to bind properly. In addition, some residues might not be relevant to binding at all, such as loops sticking out from the binding site.

Trajectories were generated from initial coarse models of the 4447 peptide–protein complexes of the training set by perturbing the native peptide (Raveh *et al.*, 2010), rigid-body docking (Alam *et al.*, 2017), or template-based docking (Johansson-Åkhe *et al.*, 2020) (see above), and InterPepScore was trained on snapshots from these trajectories. After performance on validation data had converged, the newly trained InterPepScore was used again with Rosetta FlexPepDock as an addition to its regular scoring terms to generate new simulation trajectories for further training in an iterative manner until performance no longer improved (after three iterations). This way, the training data was kept as similar as possible to cases which the method would be run on, resulting in a form of online training where the training set is continuously updated.

Although fully differentiable with regards to vertex and edge features, graph networks are not differentiable in regards to the addition and subtraction of edges, and can as such not be used for the gradient descent calculation for the energy minimization steps in the FlexPepDock protocol. Minimization steps are included in FlexPepDock at regular intervals between Monte Carlo steps if the total score of the complex is too poor as compared to a previous state, to solve potential clashes introduced. Analysis on validation data showed that the minimization step did not contribute to the final quality of refined models when InterPepScore was included in the refinement. As the minimization steps would not take InterPepScore into account while the other Monte Carlo-based steps would, it is probable that including the minimization steps would lead to situations in which it worked against the rest of the protocol. The lack of differentiability with regards to edge addition or subtraction does not affect the non-minimization parts of the protocol and the conformational space sampled by the protocol is not limited by the removal of the minimization steps (Supplementary Fig. S1) even though the overall number of accepted Monte Carlo cycles decreases by 31.3% on average.

In addition to being used for early stopping (stopping training when performance on validation data ceases to improve), the performance on the validation set was also used to find optimal hyperparameters regarding features such as degree of dropout, whether batch normalization was needed during training, the number of graph network layers, which activation function to use, the weight with which to include InterPepScore in the scoring function, and whether using the BLOSUM62 matrix or a pretrained embedding for protein sequence such as Bepler (Bepler and Berger, 2019) or ProtBert (Elnaggar *et al.*, 2020) would be optimal as vertex features. In general, more graph network layers and more expressive vertex features yielded minor improvements to performance on training data and validation data at first, but would quickly result in massive overtraining, probably stemming from the restrictive number of truly unique interaction complexes and sequences in the training set.

## 3 Results and analysis

### 3.1 InterPepScore improves refinement

The performance of the FlexPepDock refinement protocol when including the InterPepScore scoring term was analyzed on the test set of 109 non-redundant peptide–protein complexes that shared no CATH superfamily annotation with any complexes from the training or validation sets. For each complex, starting points were generated the same way as for the training data. For the test and validation sets, each scheme for generating starting points contributed with 4 starting models, in total 12 starting points per complex. The FlexPepDock refinement protocol was run 200 times with and without InterPepScore for each starting point. In all cases, the receptor was relaxed and the exposed side-chains perturbed prior to docking to simulate an unbound-to-bound docking scenario.

**Fig. 2.**
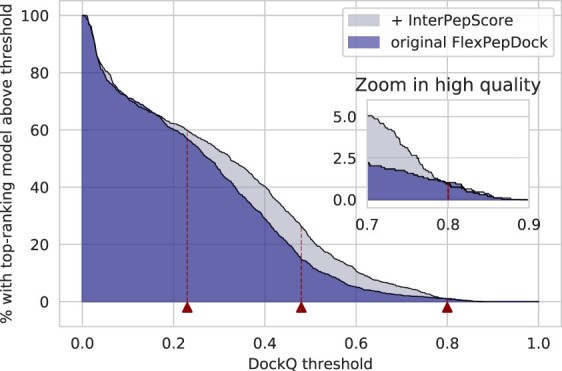
For the 109 peptide–protein complexes investigated, the addition of InterPepScore to the FlexPepDock refinement protocol both during folding and final decoy selection consistently improves the quality of the final selected decoys as measured by DockQ score (Basu and Wallner, 2016). With InterPepScore, the top-scoring decoy achieves a DockQ-score of at least Medium quality (>0.49) in 26.1% of all cases, as opposed to only 14.8% without. The DockQ cutoffs for Acceptable, Medium and High quality have been marked with red triangles (A color version of this figure appears in the online version of this article.)

By including the InterPepScore both in the sampling and selection stage of the refinement the DockQ score is improved (Fig. 2), resulting in more models of Acceptable and Medium quality. The improvement is largest for the Medium quality where the original FlexPepDock protocol produced models with a DockQ indicating Medium quality model or better for 14.8% of the targets, which improved to 26.1% of the targets using InterPepScore. For High quality there is no difference, but only around 1% of the models have that quality.

**Fig. 3.**
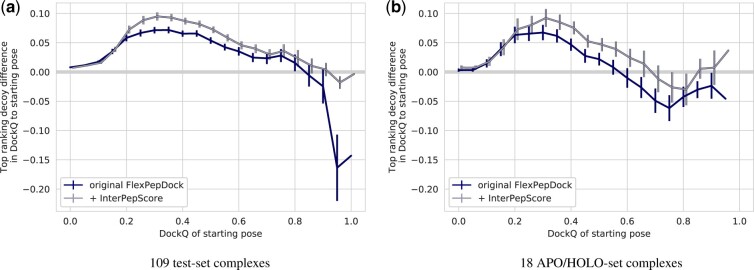
For each starting position for each of the 109 peptide–protein complexes of the test set, the addition of InterPepScore to the FlexPepDock refinement protocol both during folding and final decoy selection consistently improves the quality of the final selected decoys as measured by DockQ score (**a**). One model is selected per starting position. Similar improvements can be seen for all but already high-quality models on the unbound-to-bound refinement set of 18 complexes (**b**)

The original FlexPepDock refinement protocol already consistently improves the quality of all decoys except those with high DockQ scores, but with InterPepScore the improvement is larger and maintained even for decoys with already excellent starting DockQ (Fig. 3). InterPepScore not only aids in final selection, but also leads to FlexPepDock producing higher quality models overall (Fig. 3). As suggested in Raveh *et al.* (2011), the *reweighted_sc* score term was used for evaluation of relaxed structures. Tests on validation data suggested small negative impact on performance when selecting models based on *total score* instead (data not shown).

### 3.2 Starting from APO state receptors

All side-chains conformations in the test set were rebuilt before docking and the receptors were relaxed *without* the peptide. However, this might not adequately model potential backbone changes some receptors undergo upon binding a peptide. To test if InterPepScore aids in refinement in true unbound-to-bound refinement cases as well, a second test-set was constructed from a list of solved structures with annotated bound and unbound conformations, provided by the creators of AutoPeptiDB (London *et al.*, 2010). After removing all complexes where the receptor shared CATH superfamily annotation with the training or validation set, 18 complexes remained for use in an *APO/HOLO-test set*. Starting positions were generated for these complexes using the same scheme as for the original test set (see above).

Overall, both the original FlexPepDock protocol and with added InterPepScore manages to successfully refine the majority of starting positions with similar performance as for the larger test set, although both methods seem to struggle to refine high quality models (Fig. 3). In addition, including InterPepScore still aids in the final quality of decoys, increasing the fraction of cases when a model of at least Medium DockQ-score quality is generated and selected from 38.1% of cases to 47.6% of cases. Lastly, for neither FlexPepDock nor FlexPepDock with InterPepScore where there any significant correlation between differences in conformational change between the unbound and bound states of the receptor and the average difference in DockQ-score to the starting position (*P*-values > 0.21), further indicating that the method is generalizable to realistic test cases. The differences in conformation were measured as the TMScore or RMSD of the entire receptor or the TMScore or RMSD of only residues involved in peptide binding (still superpositioning on the entire receptor).

Note that while this test might be closer to a realistic case, it is important to remember the small size of this secondary test set and that differences might not be statistically significant. In addition, peptides from this set are on average smaller than those from the larger test set (9.5 residues on average as opposed to 13.5), which might make the refinement simpler.

### 3.3 Is InterPepScore biased toward certain folds?

To investigate if InterPepScore is biased toward certain CATH superfamilies, the distributions of differences in DockQ-score between refined models and their starting positions (ΔDockQ) were investigated in two ways.

First, the influence of individual CATH superfamilies on the analyses at large was measured by analyzing if the distribution of ΔDockQ changed significantly when each superfamily was removed from the test set one at a time, as analyzed through two-sided *t*-test and Kolmogorov–Smirnov. In all cases the *P*-values where >0.4, indicating that if there is any bias toward any superfamily, that bias is not enough to pose a significant difference to the overall results.

Next, a two-sided *t*-test per CATH superfamily was made to analyze if the means of ΔDockQ of any single superfamily were significantly different from the mean of the distribution of the rest of the test set. Here, it seems as InterPepScore has some biases, i.e. it perform relatively better or worse for some superfamilies, as does FlexPepDock refinement without InterPepScore, see Table 1. Interestingly, the addition of InterPepScore introduces a new negative bias against a superfamily (3.10.20.90) that the original FlexPepDock had no bias against, and strengthens the already existing negative bias toward 1.10.238.10. In addition, InterPepScore also positively biases toward three other CATH superfamilies.

**Table 1.** CATH superfamilies with significantly different means of differences in DockQ to starting positions (Δ*DockQ*) as compared to the rest of the test set

CATH annotation	Statistic	*P*-value
+InterPepScore		
1.10.238.10	−3.71	2.18e^–4^
1.20.920.10	2.56	0.0106
2.60.40.150	4.59	5.02e^–6^
2.60.210.10	2.36	0.0186
3.10.20.90	−2.13	0.0330
Original FlexPepDock		
1.10.238.10	−3.30	0.0010
1.10.246.190	−3.67	2.57e^–4^
2.20.70.10	3.30	9.84e^–4^

The CATH superfamilies with significant biases were investigated further to see if any general conclusions could be drawn regarding InterPepScore behavior and bias. The CATH superfamily 1.10.238.10, with the largest negative bias, is EF-hand folds, which are small bundles of α-helices connected by Calcium-binding loops. This is a fold that both original FlexPepDock refinement and the version improved with InterPepScore struggled with. Since all co-factors and solvent molecules were removed in the tests in this study, these structures lacked the Calcium atoms normally present, which could have negatively affected the scoring of the structure.

The positive bias introduced by InterPepScore toward 1.20.920.10, a superfamily containing histone-binding proteins, also seems to stem from the design of the dataset, which was redundancy-reduced with regards only to the receptor and not the peptide. As most peptides in the dataset are small, only the receptors were used to filter out homologs between the sets, as significant sequence matches between peptides would be impossible without extensive review of their studies of origin to find the sequences the peptides were exercised from. The histone interaction network includes many interactions to histone N-terminal regions which are often examined in the form of peptide–protein interactions, some of which are present in the training set.

**Fig. 4.**
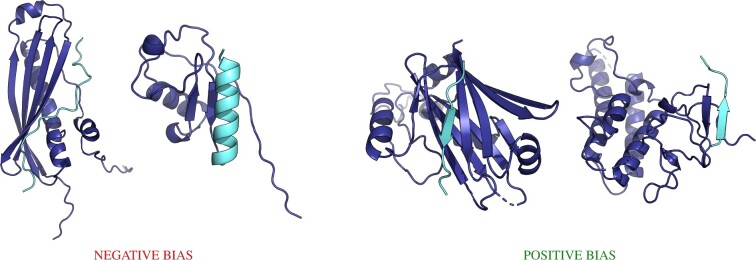
Examples of complexes which are part of CATH superfamilies toward which InterPepScore introduces significant biases. The two leftmost structures are part of superfamily 3.10.20.90, consisting of complexes where the peptides mostly interact with sheet structures through other modes of binding than β-sheet reinforcement, and toward which InterPepScore introduces a negative bias. The two rightmost examples are from CATH superfamilies where peptides mainly interact through β-sheet reinforcement, and toward which InterPepScore introduces a positive bias (2.60.210.10 and 1.20.920.10). In all images, the receptor is colored dark blue and the peptide light cyan (A color version of this figure appears in the online version of this article.)

More interesting, perhaps, are the other superfamilies for which InterPepScore introduced significant negative biases. 3.10.20.90 is the ‘Phosphoatidylinositol 3-kinase Catalytic Subunit; Chain A, domain 1’ superfamily, belonging to the ubiquitin-like (UB-roll) topology. Although the binding modes for the peptides in this superfamily are quite varied, most of them interact with a β-sheet in the receptor, but not through sheet reinforcement. Rather, the peptide adopts various non-sheet structures, such as in the form of a helix in contact with the side-chains of a two-strand sheet or an extended loop making spurious β-sheet reinforcement-like contacts but with tight loops and turns along itself. In the superfamily 2.60.210.10, toward which InterPepScore has a positive bias, interactions occur through classic sheet reinforcement, indicating InterPepScore does not simply bias against interactions through sheet structures, but rather is perhaps biased against uncommon sheet interactions. Examples can be found in Figure 4.

**Fig. 5.**
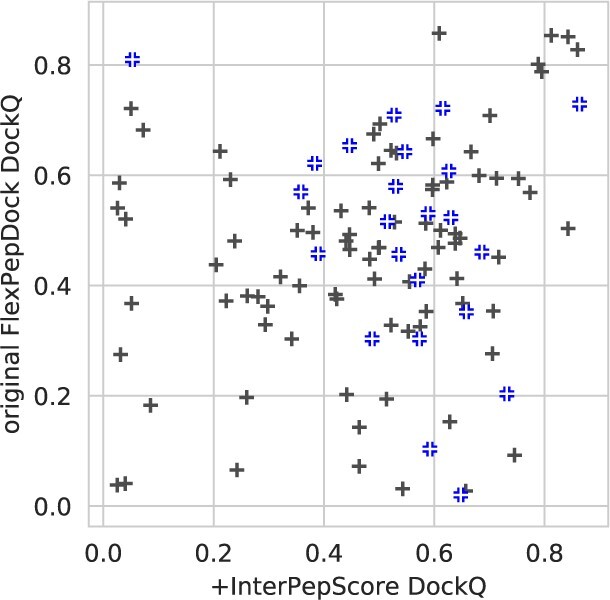
For each complex, the difference in DockQ score for the best of the top 10 final refined models as selected by standard FlexPepDock and the InterPepScore enhanced version. Each point represents one complex. Complexes where the peptide interact through β-sheet reinforcement are marked with white crosses with blue border (A color version of this figure appears in the online version of this article.)

When comparing biases, it could be interesting to analyze the complexes for which the final selected refined models differ the most between FlexPepDock protocol with and without InterPepScore. As can be seen in Figure 5, there exists many complexes for which either method performs better. Marked with blue in the figure are the complexes in which interaction is mediated via β-sheet reinforcement, which shows the positive bias introduced by InterPepScore toward such interactions but with one clear outlier: the complex for which there is the largest difference in favor of original FlexPepDock. While this complex does indeed interact through β-sheet reinforcement, it is also a structure reliant on zinc co-factors, a kind of structure which InterPepScore worsens the already inherent bias against. The complex for which the difference is largest in the opposite direction consists of a particularly long α-helical peptide of 24 residues. This bias of peptide size seem to be a general rule. For the cases in which the original FlexPepDock refinement protocol failed to create and select an Acceptable model but the InterPepScore-enhanced protocol succeeded, 59.3% of the peptides had a length of at least 15 residues (Supplementary Fig. S4). This can be contrasted with the fact that only 38.7% of the peptides in the dataset overall have at least this length.

As such, the authors do not recommend including InterPepScore when running the FlexPepDock refinement protocol on models of complexes believed to interact through uncommon β-sheet interactions. In general however, the addition of InterPepScore should improve the performance, especially in cases where it us suspected the interaction is mediated through canonical β-sheet reinforcement or for longer peptides.

### 3.4 InterPepScore refines AlphaFold models

**Fig. 6.**
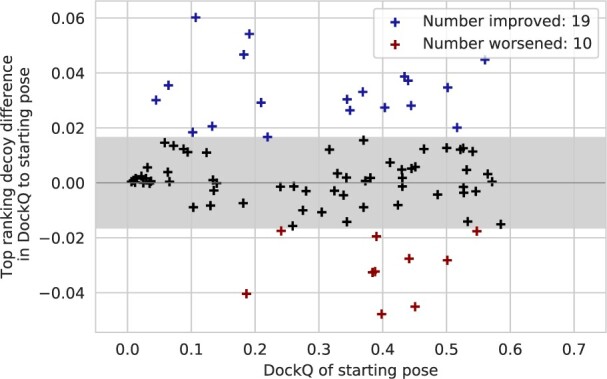
The overall change in DockQ-score for the top scoring refined models as compared to their starting positions generated by AlphaFold-Multimer. The gray shaded area shows the boundary of two standard deviations from zero. Note the decreased magnitude in DockQ differences from starting points as compared to the other test sets

To demonstrate general functionality of InterPepScore in refinement, we generated starting models for the test set using AlphaFold-Multimer (Evans *et al.*, 2021), and applied the refinement to these starting models as for the other tests. The starting models generated by AlphaFold-Multimer are already refined and of high quality, with few or no clashes, more satisfied bond angles and hydrogen bonds and less non-interacting residues, although they are not necessarily of higher DockQ. This makes the refinement problem harder, as some simple improvements such as shifting the rest of the peptide into binding or resolving clashes are not available. Indeed, although this test demonstrated a much more modest overall model improvement compared to their starting points (Supplementary Fig. S5), with changes most often only within 0.02 DockQ score, the InterPepScore still enhanced FlexPepDock refinement, significantly improving the DockQ-score for 19/109 complexes in the test set, while 10/109 complexes got significantly worse DockQ-score (Fig. 6). Difference greater than two standard deviations from zero was considered significant. FlexPepDock refinement without InterPepScore only improved 13/109 complexes significantly, while 7/109 complexes got significantly worse (data not shown).

The same test was also performed on starting positions generated by AlphaFold monomer version with a 30 glycine linker as in Tsaban *et al.* (2022). Even though the improvements in absolute terms are small and most refinements (85/109) do not change the DockQ score significantly, just as the case of AlphaFold-Multimer, InterPepScore still improved 19/109 targets significantly, while only 5/109 targets got significantly worse (Supplementary Fig. S6).

### 3.5 Reduced runtime with InterPepScore

As the minimization steps of the FlexPepDock refinement protocol were skipped when including InterPepScore as a scoring term, the protocol could theoretically run faster with InterPepScore, enabling greater sampling in the same time by running more iterations of the protocol. Indeed, the skipping of the minimization steps does slightly decrease the computational time required for the protocol, even with the time added for calculating InterPepScore (Supplementary Table S2), keeping the runtime of InterPepScore enhanced FlexPepDock similar to that of the original protocol. However, the scaling of InterPepScore for larger complexes is better than the minimization steps, meaning that InterPepScore is relatively faster for larger complexes.

When discussing runtime, the deciding factor in the end is how many times the protocol needs to be run before reliably converging on a stable sampled minimum. That is, after how many runs can the user be certain the method has sampled the best conformation it will select. To analyze this, a subset of 15 complexes with different CATH superfamilies from the larger test set were randomly selected and the protocol was run repeatedly to generate 20 000 models for each of these complexes, both with and without InterPepScore. Although InterPepScore-enhanced reweighted_sc remained the selection criteria with highest correlation to DockQ score, and thus optimal when selecting a large number of models (as in preparation for clustering), the interface score (I_sc) proved slightly better for selecting the best model for both FlexPepDock with and without InterPepScore when a large number of models were generated (average DockQ difference to best model sampled of 0.29 versus 0.33).

With InterPepScore, it took 13 400 runs to reliably sample and select the top decoy for 90% of the complexes as contrasted with 14 600 runs for FlexPepDock without InterPepScore. The average DockQ scores of these top decoys were 0.504 and 0.446, respectively. Thus, including InterPepScore leads to less computational time needed overall, as time taken to run the protocol a single time remains largely unaffected while it can be run approximately 1000 fewer times and still reliably reach optimal results.

## 4 Conclusion

InterPepScore is easy to use with pyRosetta and contributes significant improvements toward the FlexPepDock refinement protocol. In general, the score-term causes the protocol to produce better structures as measured by DockQ-score. In some cases, for example uncommon β-sheet interactions, InterPepScore introduces negative biases in performance, while introducing positive biases for other cases, such as classic β-sheet reinforcement.

InterPepScore serves as a proof-of-concept that machine learning regressors can be used in combination with statistical potentials and first principle energy functions, and can be trained to integrate into docking protocols based in these. Future works may explore additional network architectures and input features, perhaps moving to an architecture with support for edge prediction to enable differentiability in regards to edges and thus making the regressor compatible with energy minimization steps of the refinement protocol.

It is our hope that many of the state-of-the-art peptide–protein docking pipelines which make use of the FlexPepDock refinement protocol will also make use of this additional scoring term to improve their refinement steps.

## Funding

This work was supported by SeRC, VR 2020-03352, CTS 20:453. Computations provided by SNIC, KAW and LiU through NSC002E.

*Conflict of Interest*: none declared.

## Supplementary Material

btac325_Supplementary_Data
