# Supplementary material for: InterPepScore: a deep learning score for improving the FlexPepDock refinement protocol
Source: Bioinformatics. 2022 May 16;38(12):3209–15. doi: 10.1093/bioinformatics/btac325 (PMC9191208; doi:10.1093/bioinformatics/btac325)
Supplement: btac325_Supplementary_Data [file btac325_supplementary_data.pdf]

Supplementary Information
for
InterPepScore: A Deep Learning Score for Improving the FlexPepDock Refinement Protocol

Isak Johansson Åkhe   Björn Wallner

April 29, 2022

1 Sequence Embeddings

Embeddings of protein sequence can achieve stronger performance than simply using BLOSUM62 columns given enough training data (ElAbd *et al.*, 2020). However, the number of unique sequences in the training set of this study was too low to train a reasonably generalizable embedder. There exists several pre-trained embedders of protein sequence which have previously been used to great effect in protein structure prediction tasks through transfer learning, such as the embedding of Bepler and Berger (Bepler and Berger, 2019) or ProtBert (Elnaggar *et al.*, 2020). Different versions of InterPepScore were trained using the outputs from these embeddings as vertex features rather than the BLOSUM62 matrix column. Indeed, using these features did lead to better performance on the training data, overfitting occured earlier resulting in higher validation loss compared to BLOSUM62, even with highly aggressive regularization (Table S1).

Vertex Features	Training Loss	Validation Loss	R to Validation
BLOSUM62	0.029	0.036	0.469
ProtBert	0.027	0.045	0.311
Bepler	0.028	0.038	0.461

Table S1: Loss of InterPepScore trained with different vertex features on the training and validation data as well as correlation between prediction and correct values on validation data (at early stopping points). In each case, the best combination of hyper-parameters found was used.

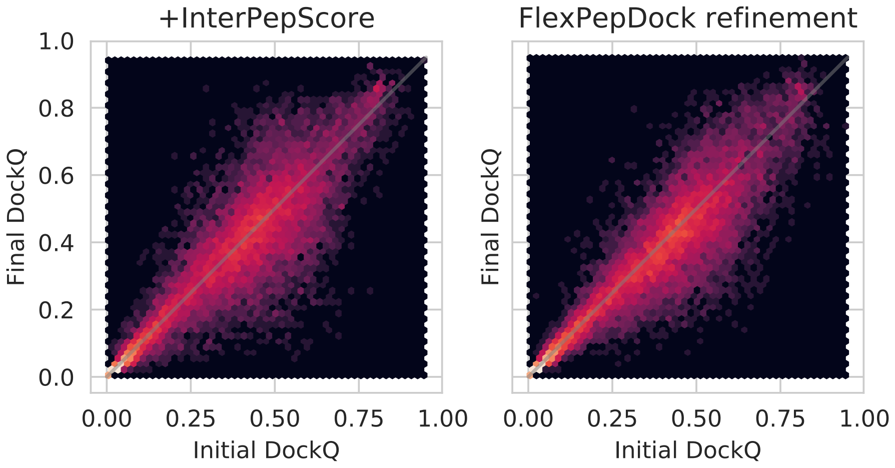

Figure S1: Hexagon density plot of refinement results. For every single run of FlexPepDock for every starting position (not only the top runs), the DockQ-score of the decoy produced compared to its starting position. The density color-gradient is log-scaled.

2 Extended Analysis

FlexPepDock with or without InterPepScore frequently generates decoys with worse DockQ-scores than their starting positions, when all generated decoys are considered, and not only the top decoys selected using the best scores, Figure S1. This is expected as FlexPepDock is a Monte-Carlo based approach which often end up in local minima or unfavorable positions, requiring the protocol to be run many times and relying on the scoring function to select structures similar to a native structure. Also visible in the same figure is the fact that FlexPepDock refinement with InterPepScore produces structures with on average higher absolute difference in DockQ as compared to their starting structures than FlexPepDock without InterPepScore does: with an absolute difference of 0.059 on average compared to 0.053.

Comparing InterPepScore to the reweighted score of FlexPepDock; since InterPepScore evaluates on the same scale independent on protein complex specifics, it also correlates better with the final DockQ of the peptide complex (correlation R: 0.394), compared to the reweighted score normalized per target (correlation R: 0.184).

Figure 5 of the main paper shows the correlation between DockQ of final top 1 selected models per complex generated by FlexPepDock with and without InterPepScore. The correlation in DockQ between the best models among top 10 generated per starting position is shown in Figure S2. The largest improvement in DockQ score is when the DockQ for InterPepScore is around and some above 0.5.

runtime (seconds) for...	original FPD	**FPD+IPS**	FPD+IPS with minimizers
average complex	74.4	**68.5**	102.8
median complex	64.5	**59.4**	90.7
60 res. complex	**24.0**	33.3	40.0
479 res. complex	220.8	**163.5**	282.9

Table S2: Runtimes of the FlexPepDock refinement protocol (FPD) with and without InterPepScore (IPS) and minimization steps. All tests run on a single core of an Intel Xeon Gold 6130 processor.

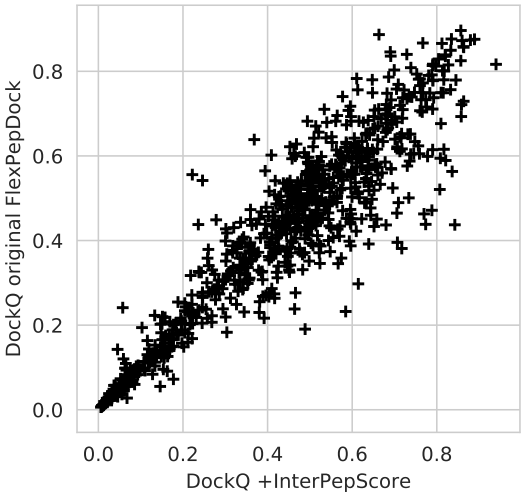

Figure S2: Correlation in DockQ between best of top 10 models generated per starting position for FlexPepDock with and without InterPepScore.

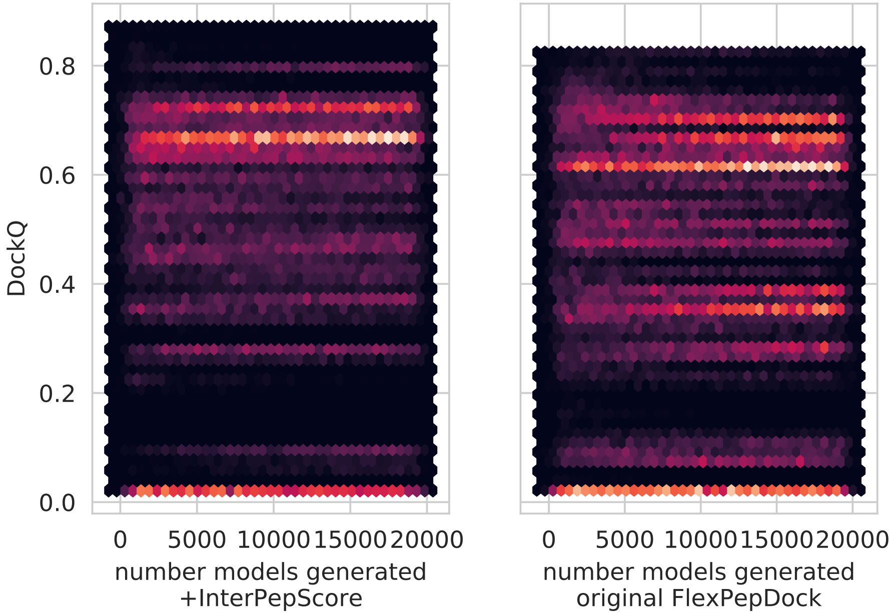

Figure S3: The distribution of the DockQ values of the top models generated with FlexPepDock with and without InterPepScore, for different numbers of models generated. At each number of models generated, a different random selection of that many models of the total 20 000 generated were evaluated (per complex). Interface Score (I_sc) was used, as it showed slightly better performance than reweighted score (reweighted_sc) when selecting top models from larger sets.

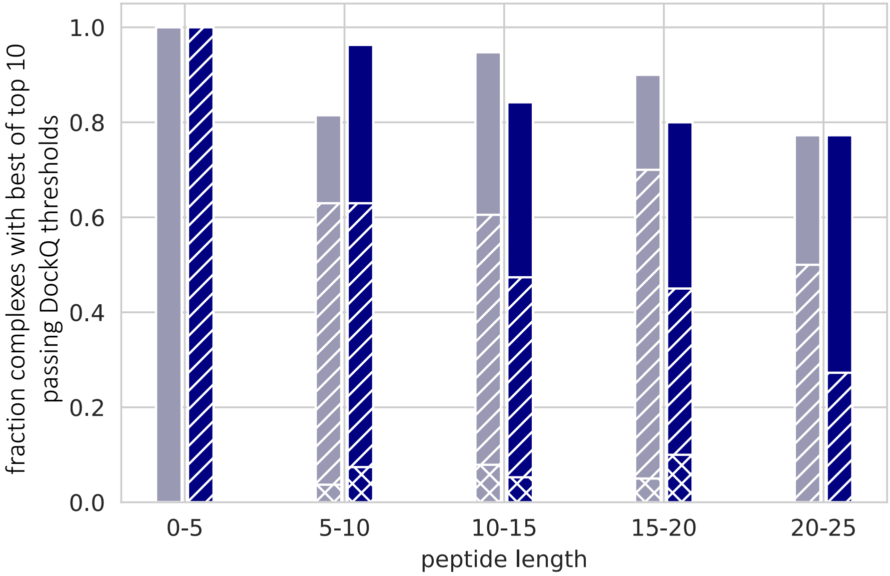

Figure S4: Fraction of complexes where the best of top 10 models has a DockQ value over the thresholds for 0.23, 0.49, and 0.80 corresponding to acceptable (whole color), medium (striped) and high quality (crossed), respectively. The left-aligned columns with lighter coloring denote FlexPepDock with InterPepScore, while the darker, right-aligned, columns denote FlexPepDock without InterPepScore.

2.1 Increased Sampling for 15 targets

For 15 randomly selected targets of the 109 of the larger test set, FlexPepDock with and without InterPepScore was run 20 000 times to analyze differences in runtime and at what point results converge. The distributions of DockQ over different number of models generated can be found in Figure S3.

2.2 Length of Peptide

The addition of InterPepScore seems to have a larger positive influence on complexes with larger peptides, Figure S4. In this figure, it is evident that larger peptides are more difficult to model for regular FlexPepDock refinement, and that peptides around length 15-20 sees the largest improvement from addition of InterPepScore.

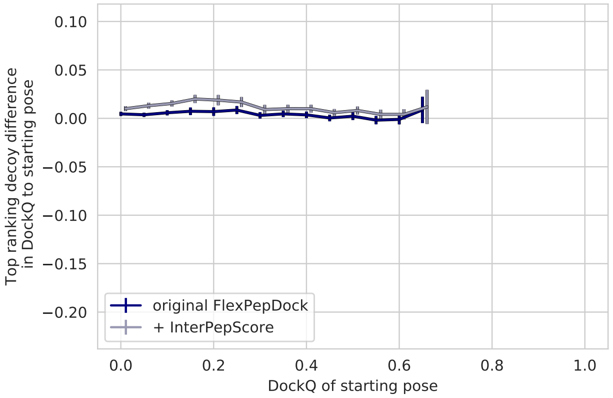

Figure S5: For each starting position for each of the 109 peptide-protein complexes of the test set, the addition of InterPepScore to the FlexPepDock refinement protocol both during folding and final decoy selection consistently improves the quality of the final selected decoys as measured by DockQ score, even for decoys generated by AlphaFold-Multimer. Note the decreased magnitude in DockQ differences from starting points as compared to the other test sets.

3 FlexPepDock refinement with InterPepScore of AlphaFold2 models

To test the applicability of FlexPepDock refinement with InterPepScore on models generated by the state-of-the-art simultaneous folding and docking protocols, it was run on models generated by AlphaFold-Multimer-v1 (Evans *et al.*, 2021), run without access to structural templates, the results of which can be found in the main paper Figure 4. The top model only, as ranked by AlphaFold's *ranking_score* was used for each complex. Figure S5 shows the average differences in comparison to the starting positions.

A similar test was also run using the docking approach including AlphaFold2 for monomers proposed by (Tsaban *et al.*, 2021). This docking involves using a polyglycine linker between the receptor and peptide to submit the complex to the AlphaFold2 inference step as one single protein chain. As can be seen in Figure S6, using FlexPepDock refinement with InterPepScore improves the quality of these models much like for the true AlphaFold-Multimer structures (Figure 5 of main paper).

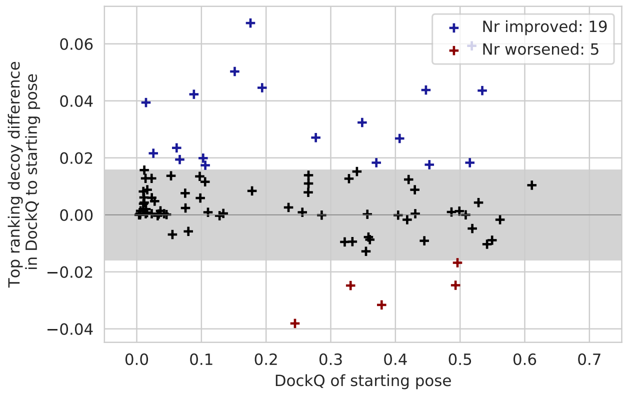

Figure S6: The capacity for FlexPepDock refinement including the InterPep-Score score term to improve the quality of models created by using AlphaFold2 with a polyglycine linker as proposed in Tsaban *et al.* (2021). Points outside the shade area are significantly (>2 standard deviations from 0) changed, positive differences are improved relative to the starting pose.

References

Bepler, T. and Berger, B. (2019). Learning protein sequence embeddings using information from structure. *arXiv preprint arXiv:1902.08661*.

ElAbd, H., Bromberg, Y., Hoarfrost, A., Lenz, T., Franke, A., and Wendorff, M. (2020). Amino acid encoding for deep learning applications. *BMC bioinformatics*, **21**(1), 1–14.

Elnaggar, A., Heinzinger, M., Dallago, C., Rihawi, G., Wang, Y., Jones, L., Gibbs, T., Feher, T., Angerer, C., Steinegger, M., *et al.* (2020). Prottrans: towards cracking the language of life's code through self-supervised deep learning and high performance computing. *arXiv preprint arXiv:2007.06225*.

Evans, R., O'Neill, M., Pritzel, A., Antropova, N., Senior, A. W., Green, T., Žídek, A., Bates, R., Blackwell, S., Yim, J., *et al.* (2021). Protein complex prediction with alphafold-multimer. *Biorxiv*.

Tsaban, T., Varga, J., Avraham, O., Ben-Aharon, Z., Khramushin, A., and Schueler-Furman, O. (2021). Harnessing protein folding neural networks for peptide-protein docking.
